# Patient perspectives on cost and quality measures in value-based cancer care

**DOI:** 10.1093/haschl/qxag040

**Published:** 2026-03-03

**Authors:** Alan Balch, Michael Lahm, Kimberly D Brunisholz, Courtney Morrow, Christine Brittle, Rebecca Bissell Genin, OluYemisi Falope, Tom Valuck

**Affiliations:** Patient Advocate Foundation, Hampton, VA, United States; Patient Author, Cincinnati, OH, United States; Johnson & Johnson Inc., Titusville, NJ, United States; Johnson & Johnson Inc., Titusville, NJ, United States; Evidera, LLC, Part of ThermoFisher Scientific, LLC, Waltham, MA, United States; Johnson & Johnson Inc., Titusville, NJ, United States; Johnson & Johnson Inc., Titusville, NJ, United States; Real Chemistry, New York, NY, United States

**Keywords:** value-based care, qualitative research, patient perspective, oncology, cancer, Medicare, EOM, Enhancing Oncology Model, OCM, Oncology Care Model

## Abstract

**Introduction:**

Patient perspectives and preferences are important when establishing quality and cost metrics for value-based payment (VBP) models, to ensure patient-centered cancer care and VBP incentives are aligned.

**Methods:**

Insights were gathered about value-based care (VBC) models from patients with Medicare and one of 4 common cancers (multiple myeloma, bladder, lung, and prostate) included in Medicare's Enhancing Oncology Model. This study included 4 virtual 2-hour focus groups and a 2-hour co-creation session. Aspects of cost and quality important to cancer care were identified (value is defined as the ratio of quality/cost).

**Results:**

Insights from the focus groups (*n* = 20) and co-creation session (*n* = 6) highlighted a lack of awareness about VBC and concerns about the effect of financial incentives on oncology care. Focus group participants identified shared decision-making and treatment effectiveness outcomes as the most important elements of quality. Co-creation participants highlighted a need for improved awareness about the implications of VBP incentives for oncology care decisions.

**Conclusion:**

In this exploratory qualitative research, patients with cancer expressed a need for transparency of VBC dynamics to help preserve the patient–physician relationship and their ability to receive innovative, affordable, and accessible care.

## Teaser

To understand patient perspectives on value in cancer care, this study explored real-world insights around value-based care (VBC) models among Medicare patients with cancer in the United States. Overall, there was a lack of awareness of VBC models and a perceived lack of transparency on whether their provider took part, despite most patients indicating they would want to know if value-based payments were affecting treatment decisions. Most patients cited treatment effectiveness and shared decision-making as the most important measures and aspects of quality. Patients expressed a need for transparency of VBC dynamics, education on value-based payment models, and reassurances that cost-containment efforts would not compromise care quality, the patient-clinician relationship, or their ability to receive innovative, affordable, and accessible care. Including patient perspectives is essential to achieving shared accountability and optimal health outcomes in VBC. Patients emphasized the need for a variety of stakeholder perspectives to be considered to ensure that real value is delivered, shared decision-making is prioritized, and quality is preserved, while making innovative care affordable and accessible for patients. Understanding and aligning patient values with current model components will support delivery of true VBC.

## Graphical Abstract

**Graphical Abstract:**
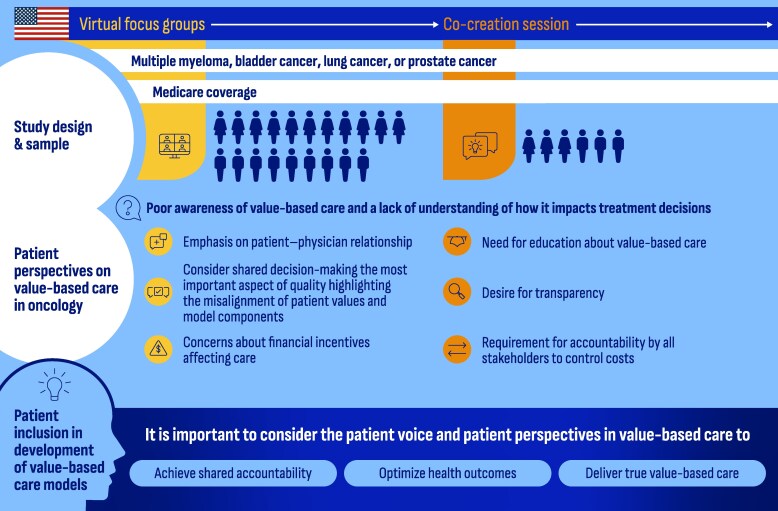

Key Takeaways

- Among Medicare patients with cancer who participated in focus groups or a co-creation session, there was a lack of awareness and a perceived lack of transparency around value-based care (VBC) models and their impact on treatment decisions.
- There was a misalignment between Medicare's Enhancing Oncology Model and patient perspectives on measures and aspects of quality, with patients in this study prioritizing treatment effectiveness and shared decision-making as key aspects of quality.
- Understanding patient perspectives and values in cancer care is essential for achieving shared accountability, optimizing health outcomes, and delivering true VBC.

## Introduction

Innovation in cancer care has contributed to major gains in cancer-related survival.^1,2^ Annual cancer-related death rates in the United States (US) declined 29% between 1999 and 2022—from 201 per 100 000 to 142 per 100 000 people^3^—and cancer survivors are now living longer.^4^ However, cancer remains a critical health concern and care costs have accelerated as patients remain on intensive treatments for long periods of time.^5^ In the United States, based on population growth, national cancer-attributed medical care costs are projected to increase to $246 billion by 2030.^5^ In addition to population growth and aging, more intensive cancer treatment, higher spending on cancer drug therapy and care, and a growing number of patients receiving treatment for longer periods contribute to increasing medical care costs.^5-7^ As payers focus on containing costs, the quality of care must not be diminished.

Healthcare value comprises quality of care relative to cost.^8^ Value-based payment (VBP) models, where providers receive payment incentives based on measures of quality and cost, are increasingly employed in primary care, with the goal of controlling costs while maintaining or improving quality of care.^9-11^ VBP models have emerged in oncology in both public and commercial payer sectors.

The Oncology Care Model (OCM) was Medicare's first value-based oncology model. Implemented by >200 practices, it ran from 2016 through 2022^12^ but was deemed unsuccessful, largely because it did not contain total costs.^13,14^ However, recent analyses suggest savings occurred among some groups, including commercially insured and Medicare Advantage patients;^15^ there was also evidence of improved care coordination and enhanced quality of care delivery, suggesting that practice transformation may be another metric of success to consider.^15-17^

In June 2022, Medicare replaced OCM with another voluntary, multi-payer model, the Enhancing Oncology Model (EOM),^18^ where practices are responsible for the total cost of care during 6-month episodes of anticancer therapy. EOM aims to reduce care costs through payment incentives and patient-focused practice redesign, including screening for health-related social needs, increased access to providers, and use of electronic health records and electronic patient-reported outcomes.^12,18,19^ Unlike OCM, where downside risk was optional, EOM includes a requirement for 2-sided risk. Practices are rewarded for performance on quality measures such as decreased avoidable acute-care utilization, patient experience, symptom management, toxicity, psychosocial health, and end-of-life care. While OCM covered most cancers and included only upside risk, EOM includes Medicare fee-for-service beneficiaries with 7 particular cancers.^12,19,20^ As of December 2025, 34 practices and one commercial payer are participating.^18^

Value-based models may significantly impact patients; it is therefore important that they understand how these models affect their healthcare. Additionally, patient perspectives should be considered when establishing quality and cost metrics for these models to ensure patient-centered cancer care and VBP incentives are aligned.^21^ However, patients are underrepresented in program design and little is known about patients’ perspectives on value in cancer care.^22-25^

This analysis aimed to address this knowledge gap. Qualitative insights were gathered from Medicare patients with cancer to understand their familiarity with value-based care (VBC) models, gain their perspectives on the aspects of quality and cost containment they view as most and least valuable, and explore their opinions on current and future program design.

## Data and methods

### Study design

This study was designed to explore patient attitudes about VBC using 4 virtual 2-hour focus groups and a 2-hour co-creation session (Figure S1) (To access the appendix, click on the Details tab of the article online). The study was exempt from institutional review board review pursuant to the terms of the US Department of Health and Human Services Policy for Protection of Human Research Subjects (45 C.F.R. §46.104(d)). Participants provided written informed consent, understood that participation was voluntary, and were compensated for their time.

### Sample

The sample was drawn from oncology members of Johnson & Johnson's Patient Engagement Research Council (PERC) program.^26-32^ The PERC comprises US residents with various chronic diseases who share their knowledge and lived experiences to inform research and promote improved understanding of patients’ needs and expectations. Patients representing various patient populations and demographics are recruited via patient advocacy groups, online advertising, and social media.

Oncology PERC members were eligible to participate in a focus group if they had Medicare coverage—either through aging into the Medicare program or having a qualifying disease or disability—so they could discuss Medicare's EOM. Co-creation session attendees were purposively invited from a subset of the focus group participants for optimal representation of the variety of viewpoints expressed in the focus groups.

### Data collection

Two-hour virtual focus groups were held (October 2024), each including Medicare patients with a mix of cancer type, race/ethnicity, and gender. The purpose was to provide information on VBC, specifically EOM, and to better understand patients’ priorities. The moderator used a semi-structured discussion guide (Figure S2).

After analysis of the focus group results, a 2-hour virtual co-creation session was held (December 2024) with a subset of patients from the focus groups to capture more in-depth perspectives. It aimed to assess patient reaction to EOM, review and validate the focus group findings, and solicit input on information patients would like to know about VBC (Figure S3).

Data collection and analysis were conducted by an independent researcher affiliated with Evidera (part of ThermoFisher Scientific, LLC) with experience and training in qualitative, patient-centered healthcare research.

### Data analysis

Qualitative analysis for the focus groups and co-creation session consisted of applying a narrative thematic analysis framework based on a priori topics. Transcripts from audio recordings of virtual sessions were reviewed and coded into a topical matrix (eg, initial impressions of VBC and feedback on specific quality topics). Using an iterative process, analytic insights were tested against new observations, and concepts were refined as they emerged. Participant quotations were used to exemplify themes. Findings were reviewed and validated by the research team (including a patient author).

### Limitations

This study has limitations. Sample sizes are relatively small and may not be generalizable to all patients with cancer, especially regarding patient populations that are younger, commercially insured, and/or of a lower socioeconomic status (SES). Self-selection bias of patients in the PERC program likely resulted in a sample skewed toward those well informed about their condition and with high education level. All participants had Medicare, which may have introduced potential bias toward more treatment. Findings reflect patient experiences, which may be subject to response and recall bias.

## Results

### Patients

Of the 64 oncology PERC members, 20 members who had Medicare participated in one of 4 focus groups, and 6 later attended the co-creation session (Figure S1). Patient characteristics are shown in Table 1.

**Table 1.** Patient demographics and clinical characteristics (October to December 2024).

Characteristic	Focus group participants (*n* = 20)	Co-creation session attendees (*n* = 6)
Number of US states represented	14	5
Age, years, median (range)	72 (53-80)	70 (53-73)
Age category, years, *n* (%)		
50-59	2 (10)	1 (17)
60-69	4 (20)	2 (33)
70-79	13 (65)	3 (50)
≥80	1 (5)	0
Gender, *n* (%)		
Female	11 (55)	3 (50)
Male	9 (45)	3 (50)
Race/ethnicity, *n* (%)		
White	14 (70)	3 (50)
Black/African American	5 (25)	2 (33)
Asian American/Pacific Islander	1 (5)	1 (17)
Highest level of education, *n* (%)		
Post-graduate	10 (50)	2 (33)
Bachelor's degree	3 (15)	2 (33)
Technical, trade, or other	2 (10)	0
Some college	5 (25)	2 (33)
Cancer type, *n* (%)		
Bladder cancer	7 (35)	1 (17)
Multiple myeloma	6 (30)	2 (33)
Prostate cancer	4 (20)	2 (33)
Lung cancer	3 (15)	1 (17)

**SOURCE** Authors’ analysis of study data.

Focus group participants had self-reported bladder cancer (35%), multiple myeloma (30%), prostate cancer (20%), or lung cancer (15%). The sample was geographically diverse (representing 14 states) and 70% of participants were White. The majority held a college degree or higher.

Co-creation session participants were largely representative of the focus group participants in terms of self-reported cancer diagnosis, race/ethnicity, age, and education (Table 1).

### Lack of awareness about VBC

Most focus group participants had not heard of VBC, and only one participant knew if their own care team participated in a value-based model. One participant said, “*Value-based care is kind of a catch-all term that doesn't really have any meaning to me.”* Another said, *“I am not familiar with this value-based model.”*

### Patient definition of value

Participants expressed concern about how value is defined in such models. They wanted to be sure that value was considered from the patient perspective.

Patients defined value as consisting of 5 core elements, including whether they are improving their chances of survival, whether they are receiving high-quality care, whether they receive care that follows best practices, whether they are getting timely care, and whether they are fully informed about their care.

One said, *“My definition of value … is, am I getting treatment that is state of the art, that's evidence-based, that's effective, the best available care? So that would be value in terms of results for me. Value in terms of cost would be, am I paying the appropriate amount for it, or is my insurance company being charged the appropriate amount for it?”* Another said, *“Value to me would be good quality care, frequent monitoring, treatments when they’re necessary. That kind of thing. That wouldn't necessarily line up with the insurance company's thoughts.”*

### Quality as component of value

**Figure 1.**
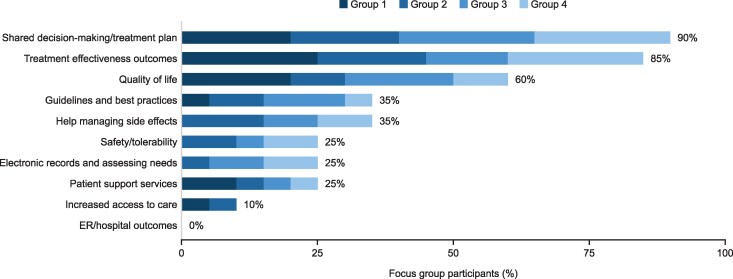
Focus group discussion (focus group participants, *n* = 20; October 2024): which of these aspects of quality are most important to you? ER, emergency room.

Patients agreed that quality is a vital component of value and had strong views on which aspects of quality were most important. Focus group participants were asked to consider 10 possible elements of quality and describe what was most important to them. Shared decision-making was cited most (90%), followed by treatment effectiveness outcomes (85%) and quality of life (QOL) (60%) (Figure 1).

Shared decision-making was seen as important because it reflects open communication between patients and providers, and because it gives patients a sense of agency and control in their care. Treatment effectiveness was seen as essential, and many patients said their main goal is survival and extension of life. One noted that it is “obvious” that treatment effectiveness is important, and participants also saw treatment effectiveness as correlated with other critical outcomes. QOL was also an important outcome measure, as QOL is a broad measure that helps to capture how they are doing in many aspects of life including physical health, mental health, and overall well-being.

### Cost as component of value

Patients were less focused on cost as a component of value than on discussions with their care team about treatment. Focus group participants were concerned that, despite having trusted relationships with their providers, cost-containment incentives of VBC models could affect their care by limiting access to evidence-based treatments. Specifically, cost guidelines may lead to some patients not getting the care they require, and providers may avoid treating high-cost patients. Participants believed VBC models with strong incentives to reduce costs could erode trust, lessening the emphasis on shared decision-making and shifting decisions from patients and physicians to third-party payers (Table 2).

**Table 2.** Participant perspectives on value-based care (focus group participants; October 2024).

Discussion topic	Theme	Representative participant perspective
**Lack of awareness and understanding of value-based care models**	Participants were not familiar with value-based care, and most did not know if their provider took part in a value-based care model	*“I do not know if my provider or providers participate in such a model. I've never heard that mentioned.* *“I've never heard these terms referred during my treatment ever.”*
	Participants felt they should know if their providers were participating in a value-based care model	*“If my provider were to participate in this value-based payment model, I would surely want to know more about it and how it would impact this ongoing care for me.”* *“I honestly don't know if my provider participates in that value-based model. And that is quite concerning to me.”*
**Patient perspectives on quality as a component of value**	Participants considered outcomes, particularly survival, to be a key indicator of quality	*“As a patient, I’m concerned about outcomes, of course, for my cancer to be in remission or under control.”* *“Quality of oncology care is primarily surviving.”* *“If I’m the patient, I want the best effectiveness and the best medicine I can get. Personally, I don't care what it costs. I want to live a couple more days.”*
	Shared decision-making was viewed as a key aspect of quality; participants emphasized the importance of trust and open communication in their relationships with their providers	*“I want to know the options of the treatment plans or opportunities for treatment that there are. I think every cancer has a number of ways you can approach it, and each one of them has their own side effects and effects on quality of life and longevity. … I want to know what my options are.”*
	Quality of life was seen as a broad indicator of quality, reflecting how patients are feeling in multiple aspects of life	*“Quality of life encapsules all these things, how you feel, how are you feeling physically and mentally, and your overall well-being. Because the better you're feeling going through your treatment process, you can better … wrap your head around the whole situation and accept what you’re going through.”*
**Patient perspectives on evidence-based care as a component of value**	Patients defined value in terms of evidence-based care, considering guidelines and best practice to correlate with effective treatment	*“I want to know that there's good research results guiding the care plan that I am following, that it's been studied, that it's shown to be effective, that the side effects are known. At this point, I really don't want to be a guinea pig.”*
**Patient perspectives on cost as a component of value**	Patients had concerns that an emphasis on cost may mean value-based care could focus on the payer instead of the patient	*“I would be real concerned about whose value we’re worried about, my value or the insurance company's value. That would certainly make a big difference.”*
	Participants felt that a focus on costs might negatively impact their care, potentially limiting access to evidence-based effective treatments	*“The thought of penalties and bonuses, I don't like that at all. That doesn't seem to me to be of any value to add to the patient. I believe that it would alter or change the way a doctor might care for a patient based off they’re going to get penalized or get a bonus.”*
	Participants were concerned a focus on cost could lessen the emphasis on shared decision-making between patients and their doctors	*“It's shifting the decision-making from doctors to Medicare. Having Medicare have more say in both what is done, what is provided, and how much it's paid for it.”*
	Participants were concerned that setting cost of care expectations based on an “average patient” might lead to some patients not getting the treatment they require	*“Am I receiving the best care that I possibly could, or are you basing it on others? … Because I know every myeloma patient, such as myself, we are all different. We do need different treatments and so forth. So I would be very concerned and wonder if they are giving me the best care.”*

**SOURCE** Authors’ analysis of study data.

### Concerns about oncology VBC models

Many patients had a strong, negative, emotional reaction to the topic of VBC. Focus group participants agreed they should be informed if their provider was part of a VBC model. One said, *“I just don't want another layer to come between the doctor's decision on what he wants to do and what's actually done.”* Another said, *“I want my doctor to think about me first. If money comes into play, I think that's a problem.”*

### Patient-designed solutions

Co-creation session attendees (*n* = 6) reviewed a summary of findings from the focus groups and detailed information about EOM based on Centers for Medicare & Medicaid Services program descriptions (Figure S3). They reconfirmed the focus group findings. They were skeptical about EOM's 2-sided risk models and felt practices may make changes to avoid financial penalties (eg, change the patients they treat or eliminate treatment choices). One said, *“I’d want the doctor making the decision based on the best information available. And if it truly is more effective … I think it's wrong not to prescribe that medication.”* Another said, *“If the quality of my care is based upon the $18,000-a-month cost of my medication, that's crazy.”* However, they generally liked EOM's required practice improvements and believed they were helpful. One said, *“I think all of these a patient should have, and it makes for a better experience for the patient.”*

Co-creation attendees stressed there is a need for improved awareness about the implications of VBP incentives for oncology care decisions. They suggested:

- Medicare and other payers should develop patient education materials about VBC. One said, “*They have to start by explaining to patients the standards that they’ve agreed to adhere to*.”
- Transparent communications from participating practices should inform patients about the risks/benefits of the models. (Note: Medicare provides a sample notification letter for practices to send to patients [https://www.cms.gov/priorities/innovation/media/document/eom-bene-notif-letter], but it focuses on practice improvements [e.g., patient navigation services], and does not include information on quality measures or 2-sided risk, and how these incentives may impact care.)^33^
  An attendee said, *“I really don't want options taken away from the people that are treating me. That's really what it comes down to. And I think the restriction on drug costs is absolutely going to limit what they're able to prescribe.”*
- Medicare should work with all stakeholders who have accountability to control drug costs. Holding providers accountable might reduce access to the most effective treatments. For example, one said, *“That should be something separate that maybe they do with the pharmaceutical industry. … Our doctors don't dictate what drugs cost.”*

## Discussion

This study gathered insights about VBC models from patients with 4 common cancers included in Medicare's EOM. It captured patients’ perspectives on aspects of cost and quality that are important in their care and explored specific topics with participants with Medicare coverage. Overall, patients had significant concerns about VBC models and raised questions about how they may affect patient care in oncology.

As members of the sponsor's existing PERC program, study participants were likely to be well-informed and active in their healthcare. They were also highly educated. However, familiarity with VBC models was low, with poor understanding of why such models were applied to cancer care, where patients emphasized their key outcomes are measured in survivability and QOL.

A misalignment was observed between how VBP models and patients with cancer define quality in cancer care. The National Academy of Medicine defines quality as the degree to which health services increase the likelihood of certain health outcomes and are consistent with current professional knowledge as measured by safety, effectiveness, patient centricity, timeliness, efficiency, and equity of the care provided.^8,34^ In this study, patients considered treatment effectiveness, shared decision-making, and QOL to be key determinants of quality; some of the measures in EOM (eg, decreased avoidable acute-care utilization) were less important to patients. Surveys have shown that patients with cancer value return to health and survival as key components of quality.^23-25^ For example, a survey of esophageal cancer survivors found the majority ranked survival and functional independence over cost.^23^

Patients emphasized the importance of their relationship with their physician and the need to protect shared decision-making and providers’ ability to deliver best-practice treatment without financial penalty. Improved transparency about how quality is defined in payment models and clarification of the underlying dynamics of VBC programs may help preserve the patient-physician relationship to uphold the shared decision-making process.

Misalignment with patient values and insufficient transparency about VBC cost benchmarks and quality metrics incentives may also contribute to poor patient understanding and raise the question of informed patient consent. While providers are required to distribute beneficiary notification letters to advise on their participation in EOM,^33^ no formal patient consent is required. Patients in the current study were largely unaware whether their provider was participating in VBC models or what it would mean for their treatment. Further, because EOM-related practice changes are often applied across the entire clinic, they may create spillover effects for patients without Medicare,^15^ highlighting the need for greater transparency about how such models may influence care for all patients.

In oncology, treatment decision-making is complex and considers the high cost of targeted diagnostics and treatment as well as the individualized patient needs. Study participants wanted their physician to make the best treatment choices, and if cost were a primary driver for treatment selection, there was concern that the physician might not be free to prescribe the best option. Moreover, they fundamentally disagreed with financial incentives targeting care cost (particularly treatment) being a primary performance metric. Concerns were voiced about barriers to obtaining effective but costly treatments, and about diminishing trust if decisions became primarily cost- vs evidence-based. Notably, however, all in the focus groups and co-creation session had Medicare coverage, which may have influenced their views about cost containment. Additionally, although income data were not available, most participants held a college degree or higher, suggesting a higher-than-average SES for this cohort. Populations with lower SES may have different perspectives on the relative importance of cost considerations, particularly as a previous survey identified a lack of financial difficulties as being the primary reason for patients not discussing costs with their treating physician.^35^ The same study also reported that approximately half of patients wanted their physician to consider costs to some degree in treatment decisions.

Another concern was the implications of setting cost expectations for broad populations at the cancer-type level in EOM. Patients voiced that financial penalties and payment incentives associated with 2-sided risk in EOM do not reward effective care and risk replacing the primacy of clinical acumen with cost. Patient access to the most appropriate yet expensive therapies may be limited, placing standard-of-care treatment and shared decision-making at risk, particularly for complex cases. It is notable that 2-sided risk in EOM has prevented risk-averse providers from joining the program.^36,37^

Insights from providers were not gathered; therefore, the study cannot validate patients’ opinions about providers’ possible reactions to payment incentives, including downside risk. However, an ongoing qualitative study among experts in oncology VBP models will report on providers’ views of current methodologies, including EOM, and potential for improvements to existing models. A second quantitative study is also ongoing to understand the aspects of quality that are most important to patients with cancer in a larger and more diverse patient sample.

## Conclusions

Patient, provider, and payer perspectives are essential for shared accountability and optimal health outcomes in VBC. In this exploratory research, patients with cancer raised concerns that VBC models may compel providers to prioritize cost over patient care and treatment effectiveness, and financial incentives might impact treatment decisions and outcomes. They stressed that all stakeholder perspectives should be considered to balance cost incentives with quality measures and ensure shared accountability. Oncology models, such as Medicare's EOM, must consult patients and preserve patient-physician relationships, shared decision-making, and quality, while making innovative care affordable and accessible.

Future research should investigate and validate concerns expressed by patients in this exploratory study, including understanding VBC perspectives in patients with and without comorbidities and with varying types of insurance coverage. Given the rapidly evolving standard of care, it is imperative that value-based models can accommodate advances in oncology care while addressing concerns associated with appropriate financial stewardship. Should value-based models fail to achieve this, there is concern for the imposition of undesirable, non-market-based solutions to contain cost. There is also an opportunity to investigate how much participation in VBC models impacts treatment decisions compared with non-participation. Other opportunities include researching how to build transparency into VBC models and how to alleviate provider risk linked to treatment decisions in cancer subpopulations with higher acuity by identifying clinical risk adjustment variables.

## Supplementary Material

qxag040_Supplementary_Data

## Data Availability

Data that support the findings of this research are not currently publicly available for sharing. Requests for sharing can be sent to the corresponding author and will be evaluated on an individual basis.
