# Supplementary material for: Patient perspectives on cost and quality measures in value-based cancer care
Source: Health Aff Sch. 2026 Mar 3;4(3):qxag040. doi: 10.1093/haschl/qxag040 (PMC12967066; doi:10.1093/haschl/qxag040)
Supplement: qxag040_Supplementary_Data [file qxag040_supplementary_data.zip › PERC Value-Based Care_RevMs_02Feb2026_CLEAN_Supp.docx]

# Supplementary Material

**Figure S1.** Study design flow diagram.


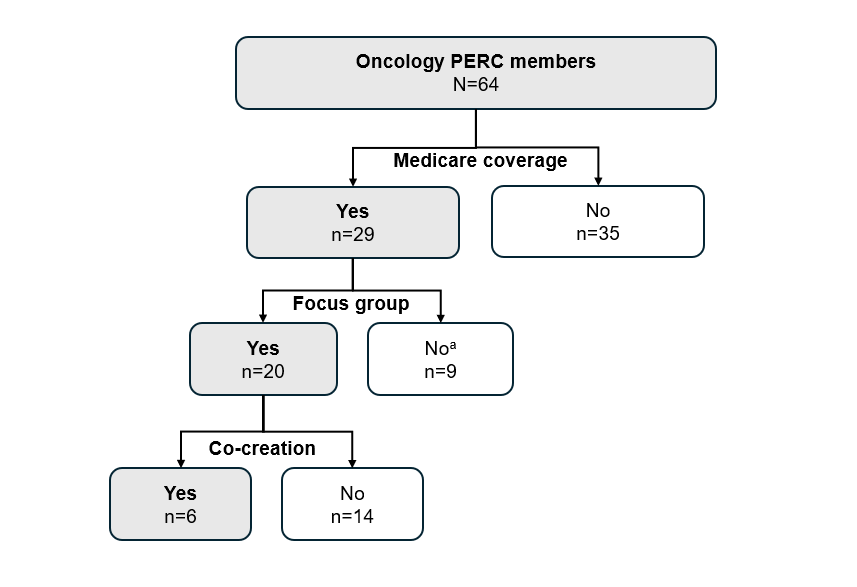


**SOURCE:** Authors’ analysis of study data. **NOTES** ^a^Did not respond to invitation to participate/were unavailable to participate in a focus group. PERC, Patient Engagement Research Council.

**Figure S2.** Focus group semi-structured discussion guide.

1. **Value-based care:** Describe value-based care and introduce **Medicare’s Enhancing Oncology Model**.

*The traditional US approach to healthcare is a fee-for-service model, where healthcare providers receive payments based on the services they provide. Many insurers are now testing value-based care models. In a value-based care model, providers receive payments based on specific results they deliver (as opposed to solely on the services they provide), as well as how they do at meeting cost-containment targets and other factors.*

*Medicare has been one of the leaders in testing these new payment models, including for oncology. Your provider may or may not participate in a model like this. Today, we will be talking more about value-based care, with the goal of understanding your thoughts and what value-based care means to you.*

- 1. Do you know if your provider participates in a value-based care model?
  2. If your provider did participate, what questions or concerns would you have?
  3. What’s your definition of “value” in oncology care? If your provider was going to be rewarded for providing high-value care, what does high-value care look like to you?

1. **Quality:** Introduce quality as an aspect of value-based care, and idea that providers may receive incentives for delivering higher quality care.

*In a value-based payment system, Medicare may require quality guidelines for your care team to follow when making treatment decisions. Providers that deliver higher quality care receive higher payments. In this section, we are going to discuss your thoughts on quality.*

- 1. What’s your definition of “quality” in oncology care?
  2. What elements of quality care do you think should be rewarded?
  3. What are the most important elements contributing to quality of care (consider shared decision-making and creating a treatment plan, patient support services, managing side effects, increased access to care, and electronic records and assessing health needs)? Why?
  4. What are the most important outcomes contributing to quality of care (consider treatment effectiveness outcomes, safety/tolerability, quality of life, emergency room/hospital outcomes, and guidelines and best practices)? Why?
  5. Review 10 areas identified in c) and d) above: If cancer care providers were going to be rewarded for providing high-quality care, which aspects of quality are the most important? Why?
  6. Are there any other aspects of quality that should be considered?

1. **Cost and payment:** Introduce cost/payment as an aspect of value-based care, and the idea that providers may be required to implement cost reductions.

*In a value-based care model, in addition to the quality guidelines we discussed, Medicare may require cost reduction guidelines for your care team to follow when making treatment decisions.*

- 1. Introduce 3 scenarios between drug A (costs more to payer) and drug B (costs less to payer), assuming: 1) both are equivalent on side effects and effectiveness, 2) drug A is more effective but equivalent on side effects, and 3) drug A has fewer side effects but is less effective.
     1. In each scenario where Medicare requires your care team to prescribe Drug B because of its lower cost, what would your reaction be?
  2. Introduce idea of 6-month time periods to calculate performance in value-based care models:
     1. What do you think of this time period to evaluate how patients are doing?
     2. Over what timeline do you typically measure your own progress?
     3. What are the benefits and drawbacks of shorter vs. longer time frames?

1. **Summary:** Revisit central question of defining quality care and reconciling cost with quality.
   1. How would you describe value-based care?
   2. How do you define value?
   3. How can the healthcare system ensure it is still providing high-quality care while also reducing the high cost of treating certain cancers?

**Figure S3.** Co-creation session semi-structured discussion guide.

1. **Medicare’s Enhancing Oncology Model (EOM):** Describe value-based care and provide details about the EOM including covered cancer types, required practice improvements, value as function of quality and cost, EOM quality measures, and EOM cost structure.

*SLIDE 1: Reminder: Value-Based Care*

*As we talked about last time, the traditional US approach to healthcare is a fee-for-service model, where healthcare providers receive payments based on the services they provide. Many insurers are now testing value-based care models. In a value-based care model, providers receive payments based on specific results they deliver (as opposed to solely on the services they provide), as well as performance meeting cost-containment targets and other factors.*

*Medicare has been one of the leaders in testing new payment models. Today, we will be talking about Medicare’s value-based care model for oncology, with the goal of understanding your thoughts and opinions about this model.*

*SLIDE 2: EOM*

*Medicare launched its value-based care model (EOM) in July 2023.*

*EOM is open to all oncology group practices. Practices voluntarily opt in.*

*Covered cancer types: high-risk breast cancer, chronic leukemia, small intestine/colorectal cancer, lung cancer, lymphoma, multiple myeloma, and high-risk prostate cancer.*

*Medicare patients remain able to choose any provider; however, the decision of whether or not to participate in EOM is made at the practice level.*

*Participating practices agree to implement a set of practice improvements (see next slide). Patients will not be charged for these services.*

*Practices will share data with Centers for Medicare & Medicaid Services* *on patient demographics and clinical status.*

*SLIDE 3: Required Practice Improvements*

1. *Provide 24/7 access to clinicians who have real-time access to medical records.*
2. *Provide patient navigation support.*
3. *Document a care plan for each patient.*
4. *Treat patients consistent with nationally recognized clinical guidelines.*
5. *Identify health-related social needs using a screening tool.*
6. *Survey patients on topics such as symptoms, functioning, behavioral health, and health-related social needs.*
7. *Utilize data for continuous quality improvement, including health equity plans.*
8. *Use certified Electronic Health Records Technology.*

*SLIDE 4: Value = Quality / Cost*

*EOM holds practices accountable for performance in two main areas:*

- *Quality*
- *Cost*

*Medicare’s evaluation of its previous value-based care model for oncology found the model had not succeeded, primarily because the model failed to reduce Medicare’s costs. The evaluation also noted that chemotherapy drug costs are one of the primary factors that increase the cost of oncology care.*

*SLIDE 5: EOM Quality Measures*

*EOM measures quality in five areas:*

- - ***Patient experience*** *(such as how patients rate their care)*
  - ***Avoidable acute care utilization*** *(such as hospital admissions or number of emergency room visits)*
  - ***Management of symptoms*** *(such as pain ratings)*
  - ***Management of psychosocial health*** *(such as screenings for depression)*
  - ***Management of end-of-life care*** *(such as admissions to hospice)*

*SLIDE 6: EOM Cost Model*

- - *Practices continue to receive typical Medicare fee-for-service payments. In addition, they will receive $110 per active covered cancer patient per month (+$30 for each patient also on Medicaid) for participating in the model.*
  - *Medicare will set expectations for what cancer care should cost, based on the average cost to treat different types of cancer.*
  - *Practices will agree to take part in two-sided risk related to cost, meaning they could earn or lose money based on performance.*
    - *Medicare will look at the actual total cost of care every 6 months for patients with active cancer covered under the model, including drug costs, and compare that to what Medicare estimates the care should have cost, with adjustments for quality of care.*
    - *Practices with high costs will pay a penalty to Medicare.*
    - *Practices with low costs will receive a bonus from Medicare.*
  1. Overall, what do you think of Medicare’s EOM? What do you like about it? What concerns do you have?
  2. What are your thoughts on required practice improvements? Do you think these improvements will meaningfully impact oncology care? Are any of these improvements more or less important to you? Is there anything you would add to this list?
  3. What is your reaction to these quality measures? Do they reflect quality? Do you think these quality measures meaningfully measure oncology care? Are any of these measures more or less important? Is there anything else that Medicare should measure to assess quality?
  4. What is your reaction to the EOM cost model? What do you think about the idea of drug costs being included in total cost of care? How, if at all, do you think holding practices accountable for total cost it might impact patient care?

1. **Patient priorities related to oncology care:** Review previous findings related to quality and cost.

*Summarized Quality Priorities from Previous Research*

***Very high:***

- - *Treatment effectiveness*
  - *Quality of life*
  - *Safety/tolerability*
  - *Shared decision-making*
  - *Access to latest therapies*

***High:***

- - *Guidelines/best practices*
  - *Help managing side effects*
  - *Cancer treatment plan*
  - *Support services (e.g., mental health, access to care)*

***Medium/low:***

- - *Electronic records*
  - *24/7 access to care*
  - *Emergency room/hospital outcomes*
  - *Patient needs assessments*
  1. Do you agree with these quality priorities? Which would be important to measure as a part of a value-based care model for oncology?
  2. What should the role of drug cost be in value-based care? What seems realistic to you? What are some practical solutions to address costs?

1. **Recommended value-based care model:** Review today’s conversation on quality and cost.
   1. Do you support the idea of value-based care for oncology? Why or why not?
   2. If a value-based care model were to exist, what should it look like? Please discuss as a group. How would it be similar to or different from Medicare’s model?
2. **Patient engagement on value-based care:** Identify what other patients should know on this topic.
   1. What should other patients know about value-based care?
   2. How would you reach patients with information on this topic?
   3. What would you tell Medicare about value-based care?
